# Social context and drug cues modulate inhibitory control in cocaine addiction: involvement of the STN evidenced through functional MRI

**DOI:** 10.1038/s41380-024-02637-y

**Published:** 2024-06-26

**Authors:** Damiano Terenzi, Nicolas Simon, Michael Joe Munyua Gachomba, Jeanne-Laure de Peretti, Bruno Nazarian, Julien Sein, Jean-Luc Anton, Didier Grandjean, Christelle Baunez, Thierry Chaminade

**Affiliations:** 1 Institut de Neurosciences de la Timone, UMR 7289 CNRS & Aix-Marseille Université, Marseille, France; 2 SESSTIM INSERM, IRD & Aix-Marseille Université, AP-HM, Marseille, France; 3 Swiss Center for Affective Science and Department of Psychology and Educational Sciences, University of Geneva, Geneva, Switzerland

**Keywords:** Neuroscience, Addiction

## Abstract

Addictions often develop in a social context, although the influence of social factors did not receive much attention in the neuroscience of addiction. Recent animal studies suggest that peer presence can reduce cocaine intake, an influence potentially mediated, among others, by the subthalamic nucleus (STN). However, there is to date no neurobiological study investigating this mediation in humans. This study investigated the impact of social context and drug cues on brain correlates of inhibitory control in individuals with and without cocaine use disorder (CUD) using functional Magnetic Resonance Imaging (fMRI). Seventeen CUD participants and 17 healthy controls (HC) performed a novel fMRI “Social” Stop-Signal Task (SSST) in the presence or absence of an observer while being exposed to cocaine-related (vs. neutral) cues eliciting craving in drug users. The results showed that CUD participants, while slower at stopping with neutral cues, recovered control level stopping abilities with cocaine cues, while HC did not show any difference. During inhibition (Stop Correct vs Stop Incorrect), activity in the right STN, right inferior frontal gyrus (IFG), and bilateral orbitofrontal cortex (OFC) varied according to the type of cue. Notably, the presence of an observer reversed this effect in most areas for CUD participants. These findings highlight the impact of social context and drug cues on inhibitory control in CUD and the mediation of these effects by the right STN and bilateral OFC, emphasizing the importance of considering the social context in addiction research. They also comfort the STN as a potential addiction treatment target.

## Introduction

Cocaine addiction, like most other addictions, takes place in a social environment that may influence drug use [1–3]. Although this influence has long been suggested by social science studies [4–6], it is only recently that neurobiological studies have started to consider the role of social factors such as the others’ presence, either as an alternative reward to substances of abuse [7–9] or as a constant part of the consumption social environment [10, 11].

Importantly, social factors may have opposite effects on drug-taking depending on the drug used such as psychostimulants (cocaine) or depressants (alcohol), but also depending on the experimental conditions. It seems indeed that in animals, the presence of peers can decrease the self-administration of morphine [12] or cocaine [11], whereas it can increase the self-administration of nicotine [13], ethanol [14], amphetamines [15], or cocaine in different conditions [10]. Interestingly, in a recent study in rats, the optimal condition to reduce cocaine consumption, compared to other peers (familiar and/or exposed to cocaine), was the presence of an unfamiliar peer naive to cocaine. Similarly, in humans with psychostimulant use disorder, similar beneficial effects of the peer presence were observed in a cross-sectional survey assessing the social environment at the moment of stimulant use [11].

Lesioning the subthalamic nucleus (STN) in rats, a small lens-shaped nucleus that belongs to the basal ganglia, potentiated the beneficial effect of peer physical presence or playback of ultrasonic vocalizations of a stranger peer, thus positioning the STN as a key neurobiological substrate of social influence on drug intake [11, 16, 17]. Furthermore, there are evidence to suggest STN deep brain stimulation (DBS) as a therapeutic strategy for addiction [18–22] which even led to one clinical attempt to date [23]. There is currently no study that has combined social context manipulations in human addiction with the study of its neural correlates.

Neurocognitive mechanisms underlying even simple behaviors are influenced by the social context in which they take place, which led to proposing the concept of second-person neuroscience [24] that advocates the “study of real-time social encounters in a truly interactive manner” [24] (page 393). This is particularly true of key brain areas involved in mentalizing, an important social mechanism [25], but is likely to extend to other systems such as the mirror neuron or reward systems [25–27]. To investigate the role of the social context on brain mechanisms of motor inhibition, we chose here to enact a second-person neuroscience situation by pretending a direct interaction with the experimenter who observes participants’ performance in real-time in half of the scanning sessions, while unbeknown to the participants presence of the experimenter was fake and controlled throughout the experiment.

In human studies, the Stop-Signal Task (SST) is one of the most used measures of impulsivity of action [28] as it requires inhibition of an overlearned ongoing action. Neuroimaging studies employing the SST have shown that the activity in the STN increases when participants successfully inhibit their response to the stop signal [29–31]. In contrast, lesioning the STN in rats leads to impaired ability to stop an ongoing action [32] and affects other forms of impulsivity of action [33]. The STN receives direct inputs from many cortical areas (the motor cortex, the inferior frontal gyrus (IFG), the orbitofrontal cortex (OFC), Anterior Cingulate cortex (ACC), constituting the so-called hyperdirect pathways. These cortico-STN hyperdirect pathways facilitate the suppression of the ongoing motor response [34–36]. Studies employing the SST in individuals with Parkinson’s disease (PD) who received STN DBS have further provided causal evidence for the involvement of the STN in successful inhibitory control [37–39].

The SST has found application in addiction research, as the ability to resist drug seeking and consumption rely heavily on the control of inhibition [40, 41]. Previous neuroimaging studies have revealed an aberrant response inhibition in addiction, which is accompanied by hypoactivations in frontal regions including the inferior frontal gyrus (IFG), the orbitofrontal cortex (OFC), and the dorsolateral prefrontal cortex (dlPFC) [42–45]. A recent fMRI study on cocaine addiction has delved deeper into investigating how drug cue salience (in the form of words) affects the modulation of inhibitory control while participants performed the SST. The findings revealed that dlPFC activity was decreased during the successful inhibition of drug (versus food) cues in CUD participants [46].

Since addictive behavior can be considered as the result of a lack of inhibitory control leading to compulsive drug use [40, 46, 47], in this study we employed a SST to specifically engage brain regions involved in inhibitory control, such as the STN and frontal cortical areas directly connected to it: the IFG and OFC. These brain regions are also involved in reward processing [27, 48, 49]. Hence, we expect that these brain areas may be strongly involved in our task and in our experimental conditions. In particular, we aimed to examine whether the social context could influence inhibitory control in individuals with cocaine addiction. To do so, we developed a novel fMRI version of the SST, namely the “social SST” or SSST. Participants with cocaine use disorder (CUD) and healthy controls (HC) performed the experimental task under two social contexts: in half of the 4 sessions, they were made to believe that they were observed by another person they could see on a video screen presenting them with the task inside the scanner (observing condition) and in the other half, no observer was present (non-observer condition). Furthermore, to induce some form of cocaine craving, we used cocaine-related stimuli (compared to neutral control stimuli) as cues indicating beginning and end of individual trials. These cues were chosen in order to trigger arousal, anticipation, and changes in behavioral motivation [47, 50–53], as well as to affect inhibitory control in drug addiction [46].

We expected that inhibitory control in CUD would be affected by both drug cues stimuli and social context and that this may be mediated by brain regions including the STN and frontal areas connected to it, in particular OFC and IFG. Moreover, we were particularly interested on the possible association between self-reported cocaine craving (CCQ questionnaire) and neural activity during cocaine-related-inhibitory control in the STN, given that previous research has indicated that inhibition of the STN can reduce motivation for cocaine or escalation of cocaine intake or compulsive cocaine seeking in rats [18–20, 22]. Additionally, we tested whether cocaine-related-inhibitory control was associated with SSRT in the task. Lastly, to explore the potential impact of other drug use severity measures, we performed correlational analyses between these measures (i.e. years of cocaine use, frequency of use in past 30 days, severity of dependence, amount of drug consumed per episode of consumption and brain activity in the STN-ROI, as well as other brain regions identified in the whole-brain analysis during cocaine-related inhibitory control (compared to neutral cues).

## Materials and methods

### Participants

The study included right-handed individuals aged 18 to 65. Seventeen participants with cocaine use disorder (CUD participants; 4 females/13 males, mean age 35.5, SD 9.23) and seventeen matched healthy controls (HC participants; 9 females/8 males, mean age 31.8, SD 13.0) took part in the study. CUD participants were recruited by author NS from the addiction unit of the Timone University Hospital in Marseille (France). All met DSM-V criteria for current cocaine addiction and underwent the Mini International Neuropsychiatric Interview (MINI) [54]. In individuals with CUD, additional substance abuse was observed with amphetamines (*n* = 1), opioids (*n* = 1), alcohol (*n* = 2), and multiple substances (*n* = 5). Craving and withdrawal symptoms of CUD participants were determined using the Cocaine Craving Questionnaire (CCQ) [55]. See Table 1. One of the CUD participants did not complete the CCQ.

**Table 1:** Demographic, clinical, and questionnaire data mean (and standard deviations) in the Cocaine Use Disorder and Health Control participants.

	CUD (*n* = 17)	HC (*n* = 17)
Gender (female)	4	9
Age (years)	35.5 (9.2)	31.8 (13.0)
Education (years)	14.5 (2.4)	16.6 (2.1)
Duration of cocaine use (years)	13.7 (7.5)	-
CCQ	28.8 (13.9)	-
DSM-V SUD	7.3 (2.3)	-
Cigarette use per day (cigarettes)	7.6 (7.0)	-
Cocaine use frequency past month (days)	10.6 (8.1)	-
Dose (grams per administration)	1.4 (1.5)	-

*CCQ* Cocaine Craving Questionnaire, *DSM-V* The Diagnostic and Statistical Manual of Mental Disorders, Fifth Edition, *SUD* substance use disorder.

Exclusion criteria consisted of (i) a history of major psychiatric or neurological disorders, (ii) MRI contraindications, (iii) the use of psychotropic drugs, and, for CUD participants, (iv) the absence of cocaine drug in urine assay. The two groups of participants were matched for demographic variables such as age (*U* = 192.5, *p* = 0.101) and gender (*χ*^2^(1) = 3.11, *p* = 0.08) but the difference for their levels of education reached significance (*U* = 72.5, *p* = 0.012). The investigator, TC, was blinded to group allocation while conducting the experiment and interacting with participants. The study was in accordance with the Declaration of Helsinki and approved by the local Ethics Committee (Comitté de Protection des Personnes Tours region center Ouest I, #2017T2-34). All participants gave written informed consent to participate in the study.

### Social Stop-Signal Task

In a standard SST, participants are instructed, in each trial, to respond as quickly as possible to a Go signal but must inhibit their response when this signal is followed by a rare Stop-signal (22.5% of trials), typically presented as an auditory tone or a visual stimulus superimposed onto the Go signal [29, 56]. In our SSST, pictures showing white cocaine powder or white chairs on black background (Coc and Neu cues) appear at the onset and disappear at the offset of each individual trial. Coc cues were chosen to simulate real situations and elicit cocaine craving in CUD subjects. Such cues are known to elicit arousal, anticipation, and impact behavioral motivation [47, 50, 57] and inhibitory control [46] in individuals with drug addiction.

**Fig. 1:**
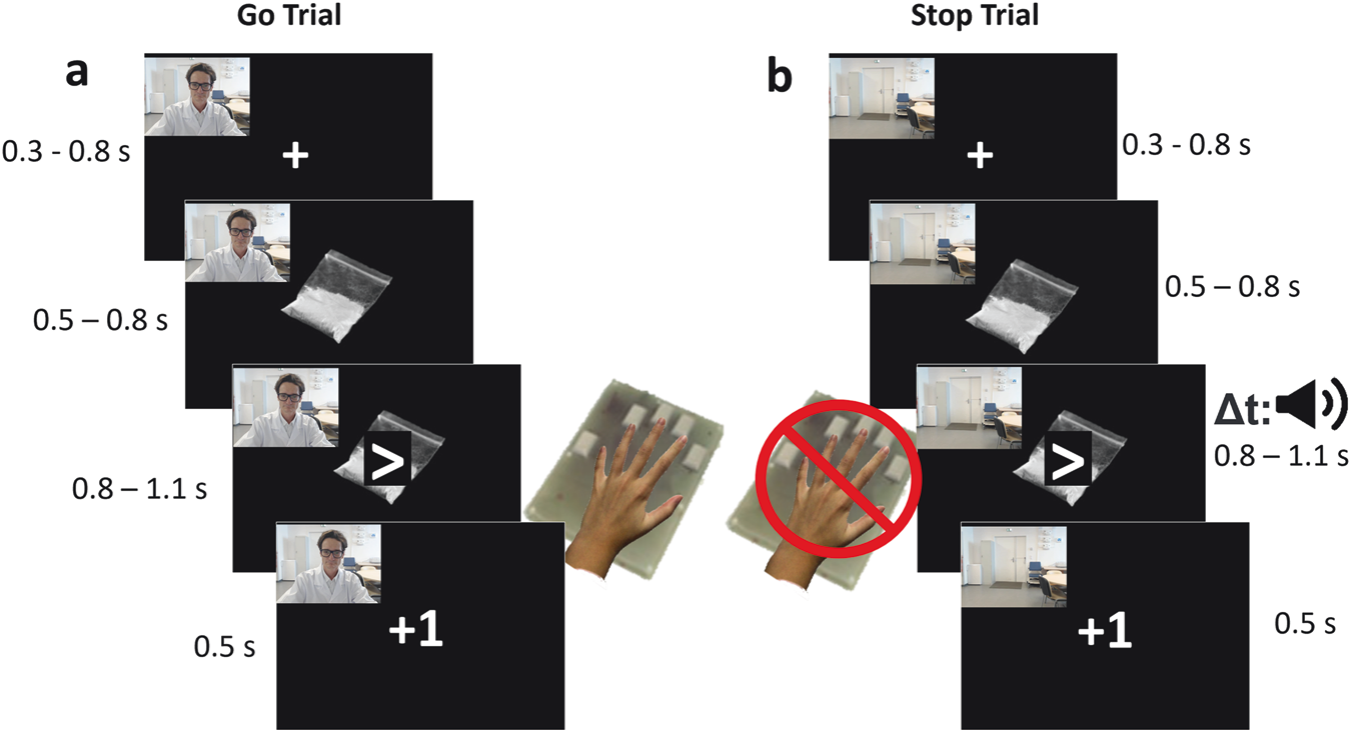
Schematic representation of the Stop-Signal Task. Each trial starts with a fixation point (+) (jittered duration between 0.3 and 0–8 s) followed by a neutral or cocaine-related cue (jittered duration between 0.5 and 0–8 s). In a Go Trial (**a**) the participant must press a button with the index or the middle finger of the right hand as quickly as possible after the presentation of an arrow pointing left or right, respectively (the maximum RT is 1.2 s). The arrow is superimposed on a cue which can be either neutral or cocaine related. Conversely, in a Stop trial (**b**), after the arrow and cue presentation, a sound (stop signal) occurs after a variable stop-signal delay (SSD) indicating the participants to withhold their response. Each trial ends with a feedback screen indicating the accuracy of the participant’s response.

During the task performed in the scanner, participants were required to press a button using their index or middle finger of the right hand as quickly as possible upon the presentation of a left or right arrow (Go trials). The arrow appeared superimposed on the cue, which could be either neutral or cocaine-related, allowing us to investigate the potential impact of cocaine-related cues on inhibitory control. During Stop trials, following the arrow presentation, a stop signal (sound) occurred after a variable delay (stop-signal delay; SSD). Participants were instructed to withhold their response upon hearing the stop signal (Fig. 1b). The SSD duration was set at 250 milliseconds and then adjusted independently for cocaine and neutral stimuli based on the participant’s success at stopping. Successful stops led to a 50 millisecond increase in the SSD duration for subsequent trials, increasing the difficulty. Conversely, unsuccessful stops resulted in a 50-millisecond decrease in the SSD duration, making subsequent trial easier [29].

Participants completed four runs of the task, with each run consisting of 12 blocks of 10 trials. Each run lasted approximately 5 min, for a total of 20 min. Each block was indicated with GO or STOP written instruction, indicating whether or not there could be stop signals occurring approximately in 20% of trials within the block. There was a total of 480 trials, with 108 Stop trials (22.5% of the trials). Importantly, to examine the impact of the presence of an observing person on inhibitory control in cocaine addiction, participants were led to believe that they could be observed via a camera by the experimenter TC wearing a labcoat in the adjacent scanner control room. In two runs they saw a visual feedback of the observing person displayed onscreen (observer condition), while the 2 other runs presented the video with an empty chair (non-observer condition). However, in reality, both videos shown to them were prerecorded to ensure rigorous control of the social context across participants. The first run always included the observer, to reinforce the cover story that explained to them that “the experimenter will observe you, especially at the beginning of the experiment, to ensure you understood and perform the task correctly”, and the order of the remaining 3 runs was randomized. See Supplementary Materials for detailed social context manipulation instructions verbatim. We did not include a control condition in which the observer is looking away to avoid a gaze-cueing like paradigm [49, 58, 59] where attention to the observer is explicitly manipulated and participants continuously look at the other’s face. In our design, they knew they were sometimes observed (via a webcam flow inlaid camera on the screen), but could not spend too much time looking directly at the observer due to the Stop-Signal Task demands (in which they had to focus on the direction of the arrow and on the stop signal).

The three main factors of the experimental paradigm are the experimental group (CUD vs. HC), an inter-subject factor, while the cue type (cocaine vs. neutral) and social context (observer vs. no-observer) are intra-subject factors.

### Data analyses

#### Behavioral data

Data were analyzed using R software version 4.1.2 (https://www.r-project.org/). Accuracy (the probability of giving a correct response) in Stop and Go trials was predicted with two separate logistic mixed effects models using the lmer function in the lm4 package [60] and explored using the Anova function type 3. Predictors consisted of the three experimental factors, group (CUD/HC), cue (cocaine/neutral), and the social context (observer/no observer). Participants’ ID was used as a random intercept. We employed linear mixed models as they can be more efficient than traditional methods when dealing with nested data or repeated measures, possibly compensating for a smaller sample size [61–64]. We further predicted Reaction Times (RT) in correct Go trials, as well as Stop-Signal Reactions Times (SSRT) in correctly inhibited Stop Trials using linear mixed models (LMMs). Predictors were the same as the ones described above. SSRT, which is the time required for one to successfully stop an ongoing response (i.e. not pressing the button) cannot be directly measured and was thus estimated using the Race model [29, 65]. More in details, RT on Go trials were rank ordered. Then, the *n*th RT was selected, where *n* is the result of the multiplication of the number of Go RTs by the probability of giving a response at a specific SSD. To get the SSRT, SSD was subtracted from this value. This process was repeated for each one of the central SSDs [66] and for each participant in each experimental condition (observer vs. non observer; cocaine-cue vs. neutral-cue). When predicting SSRT, the SSD was used as covariate in the model to account for SSD related differences in the SSRT.

#### Neuroimaging data

Data were acquired with a 3 T MRI system (Siemens Magnetom Skyra) using a 64-channel head coil. Standard procedures were employed to preprocess the fMRI data. The volumes acquired correspond to the blood oxygenation level-dependent signal (BOLD) in 2.5 × 2.5 × 2.5 cm^3^ voxels of the brain (repetition time 1.224 s). Each volume includes 54 slices of 84 × 84 in-plane voxels. The volumes were slice-time corrected, realigned on the first one, and corrected for the deformation due to the local distortion of the magnetic field and participants’ movement. Spatial normalization of the imaged brains of all participants in the standard MNI space was performed using the DARTEL procedure [67]. Individual analyses were performed for each participant and run. Functional data were spatially smoothed with a Gaussian kernel (full-width at half-maximum of 5 mm).

The following events were modeled after convolution with a canonical hemodynamic response function: Stop Correct Cocaine, Stop Correct Neutral, Stop Incorrect Cocaine, Stop Incorrect Neutral. Eventually, a final event grouped all trials that did not fit in the previous categories, such as failed go trials (e.g. answer given on the wrong side or absence of recorded click). Trials were modeled as events, with null duration and onset at the presentation of the arrow. Several nuisance covariates were calculated to delete motion and physiological artifacts using the RETROICOR method from the heart pulse and respiration monitored using a photoplethysmograph and pneumatic belt, respectively, global gray matter signal, white matter activity, and cerebrospinal fluid activity (PhysIO toolbox from the TAPAS toolkit) [68]. Single regressors represented the volumes with large movements from the participant.

To examine the neural signature of inhibitory control during the SSST, contrasts between Stop Correct and Stop Incorrect [45, 46] were computed for each Cue type (Cocaine / Neutral) and used in a second-level analysis performed with GLM-Flex. GLM-Flex toolbox (https://habs.mgh.harvard.edu/researchers/data-tools/glm-flex-fast2/) allowed the analysis of factorial designs when more than 2 factors are present, and the effects of between-participants variable Group, between-run factor Social Context and within-run factor Cue Type were modeled. The three-way interaction Group × Social Context × Cue Type was computed, while factors Participants and Sessions were used as between- and within-participant random factors respectively. To address potential movement artifacts in CUD individuals during MRI, we assessed their impact by comparing framewise displacement (FD) between the CUD and HC groups. Our findings revealed higher FD in the CUD group (*t*(32) = 2.15; *p* = 0.04). Subsequently, we adjusted for these motion artifacts by integrating motion parameters as covariates of non-interest in the GLM-Flex model, effectively eliminating their effects across all subjects. Whole-brain statistical maps were voxel-level thresholded at *p* < 0.001 before undergoing cluster-level familywise error (FWE) correction (*P*_FWE_ < 0.05), in accordance with standard practices to reduce Type I error [69]. Whole-brain tests of the individual comparisons would have suffered, from a statistical power point of view, from multiple comparisons corrections, and yield issues on the kind of procedure that can be used for such correction within the 3 dimensional voxel space. We opted for a more straightforward approach, in which the response within the clusters significantly affected by experimental factors and identified by the 3-way interaction is summarized by its mean and then subjected to a simpler linear statistical approach using the same tools as for the behavior (and more generally for behavioral data). This is not double dipping [70] as we do not aim to increase the statistical significance of the initial fMRI whole-brain analysis (the 3-way interaction, that respects all practices of multiple comparison corrections for whole-brain analysis) but to further characterize how the identified clusters, therefore considered as “wholes”, are affected by the experimental factors assessed with classical linear statistics.

## Results

### Behavioral results

#### Stop-Signal Reaction Times (SSRT) in Stop trials

**Fig. 2:**
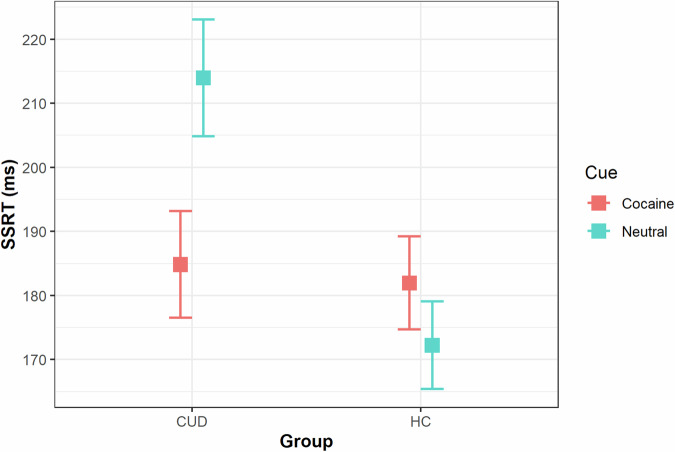
Stop-Signal Reaction Times (SSRT). CUD participants were slower at stopping (higher SSRT) than HC when presented with neutral cues but were improved (faster SSRT compared to neutral cues (*p* = 0.015)) when presented with cocaine-related cues to reach the level of the HC participants, regardless of the observer conditions. No differences in SSRT between these types of cues were found in HC participants.

According to the consensus guidelines for the SST analysis recommendations [56], we excluded one HC participant whose data violated the Horse Race model assumption of faster mean missed Stop RT compared to mean Go RT. Also, we only included data from task runs where participants exhibited a specific performance profile including a minimum Go accuracy of 60%, balanced Stop accuracy between 25% and 75%, and a positive average SSRT. This resulted in the exclusion of two additional participants (one HC and one CUD) and individual task runs from six other participants. Importantly, the number of valid task runs remaining after these exclusions did not show any significant difference between the groups (*t*(128) = −0.33, *p* = 0.745). Next, we performed a LMM investigating SSRT in correctly inhibited Stop trials. We limited our initial analyses to trials with neutral cues to maintain consistency with prior Stop-Signal Task studies not using cocaine cues. This analysis showed a main effect of Group [*χ*^2^ (1) = 6.28, *p* = 0.012]. Post-hoc analysis showed slower SSRT (reduced inhibition) for users compared to controls (*β* = −51.00, *t* = −2.63, *p* = 0.013). No other significant effects of factors emerged (all *Ps* > 0.834). When performing a second LMM including all trial types (both neutral and cocaine cues), this model led to a significant two-way interaction Group x Cue type [*χ*^2^ (1) = 5.91, *p* = 0.015]. Post-hoc analysis showed that cocaine users had faster SSRT for cocaine-related trials compared to neutral ones (*β* = −25.33, *t* = −3.00, *p* = 0.015) (Fig. 2). This result persisted even when controlling for participants’ educational level (*β*  = −25.34, *t* = −3.01, *p* = 0.015). No other significant results emerged (all *Ps* > 0.27). Notably, the results concerning SSRT remained consistent when including all the available data (see Supplementary Materials).

#### Accuracy in Stop trials

The mixed effects logistic regression model investigating the accuracy on Stop trials revealed no significant main effects of Group [*χ*^2^ (1) = 1.72, *p* = 0.189], Cue type [*χ*^2^ (1) = 0.46, *p* = 0.496], Social context [*χ*^2^ (1) = 0.49, *p* = 0.485]), nor significant interactions between these factors (all *Ps* > 0.250).

#### Accuracy in Go trials

the mixed effects logistic regression model investigating the accuracy on Go trials showed no significant main effects of Group [*χ*^2^ (1) = 1.68, *p* = 0.194], Cue type [*χ*^2^ (1) = 1.26, *p* = 0.261], Social context [*χ*^2^ (1) = 0.84, *p* = 0.358]; while the triple interaction Group × Social context × Cue type resulted statistically significant [*χ*^2^ (1) = 4.71, *p* = 0.030]. Post-hoc analysis (Tukey correction) showed no significant results (all *Ps* > 0.075).

#### Reaction Times (RT) in Go trials

The LMM for RT in correct Go trials showed a marginally significant main effect of Group [*χ*^2^ (1) = 3.82, *p* = 0.0506] and a significant two-way Group × Cue type interaction [*χ*^2^ (1) = 4.46, *p* = 0.034]. There were no other significant results (all *Ps* > 0.20). Post-hoc analysis on the main effect of Group showed that cocaine users were slower than control participants in Go trials (*β* = 45.3, *t* = 2.20, *p* = 0.028). Post-hoc analysis on the Group × Cue type interaction did not reveal significant results (all *Ps* > 0.078).

### Neuroimaging results

The second-level analysis examining the neural signature of inhibitory control with GLM-Flex (contrast: Stop Correct > Stop Incorrect) led to significant Group × Social context × Cue type interactions in the bilateral OFC, right IFG, left MTG, and left visual cortex (V1) (*F*(1, 32) = 13.12, *p* < 0.001, *P*_FWE_ < 0.05). See Table 2.

**Table 2:** Whole-brain results.

Brain regions	Side	Cluster size	Peak MNI			Peak *Z*-value	*p*-value cluster (corrected)
			x	y	z		
Orbitofrontal Cortex	Left	166	−40	54	−10	4.55	*p* = 0.016
Orbitofrontal Cortex	Right	151	40	56	−14	4.36	*p* = 0.049
Inferior Frontal gyrus	Right	133	56	27	28	4.43	*p* = 0.026
Middle Temporal gyrus	Left	252	−63	−6	−16	5.14	*p* = 0.001
Primary visual cortex	Left	222	−14	−102	−6	4.62	*p* = 0.003

Regions showing significant Group × Social context × Cue type interaction (Stop Correct > Stop Incorrect). (*P*_FWE_ < 0.05).

**Fig. 3:**
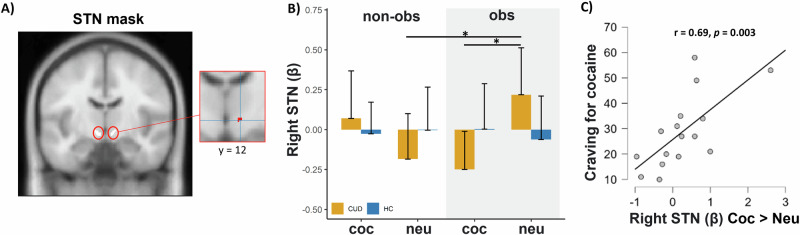
Brain activations related to inhibitory control for cocaine and neutral cues in presence or absence of an observer. **A** The left image is a bilateral STN mask defined by [87]. The right image is a zoom-in inset showing significant Group × Social Context × Cue-type interaction [*χ*^2^ (1) = 4.82, *p* = 0.028] within the right STN mask for the contrast Stop Correct > Stop Incorrect. **B** bar graph showing relative right STN activity to cocaine and neutral cues in CUD and HC participants when being observed compared to when they were not. Higher values indicate higher STN activity during inhibition. The error bars represent the standard mean of error. **C** Craving for cocaine in CUD participants is positively associated with right STN activity during cocaine-related inhibitory control (cocaine vs neutral) in the non-observer condition (*r* = 0.69, *p* = 0.003). *p < 0.05.

**Fig. 4:**
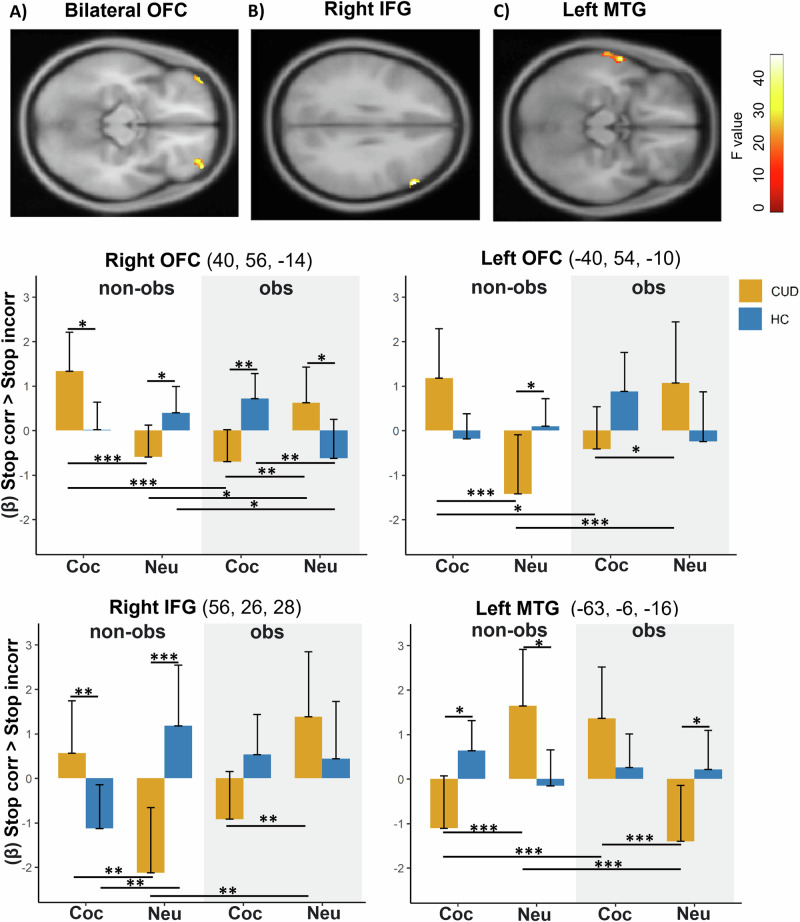
Brain activations related to inhibitory control for cocaine and neutral cues in presence or not of an observer. Whole-brain results show significant Group × Social Context × Cue-type interactions in the left and right OFC (**A**), right IFG (**B**), and left MTG (**C**) (*P*_FWE_ < 0.05) for the contrast Stop Correct > Stop Incorrect. The bar graphs represent mean signal intensity from these significant interaction clusters. Higher values indicate higher activity during inhibitory control. *p < 0.05; **p < 0.01; ***p < 0.001.

Subsequently, we ran new analyses on the extracted summary of the cluster’s activation. In particular, these significant interaction clusters were saved as separate masks using xjView (alivelearn.net/xjview/). Mean signal intensity from each cluster mask region was then extracted for each participant and task run. These values were entered into separate LMMs in R to explore which factor is driving the observed interactions. The same analysis was performed by focusing on the a priori defined ROIs in the bilateral STN. Results showed significant interactions in the right STN [*χ*^2^ (1) = 4.82, *p* = 0.028] (Fig. 3), left OFC [*χ*^2^ (1) = 14.62, *p* < 0.001], right OFC [*χ*^2^ (1) = 23.96, *p* < 0.001], right IFG [*χ*^2^ (1) = 18.57, *p* < 0.001], left MTG [*χ*^2^ (1) = 20.54, *p* < 0.001], and left visual cortex (V1) [χ^2^ (1) = 22.84, *p* < 0.001]. See Fig. 4. No significant results emerged in the left STN (*p* = 0.831).

#### CUD participants change in neural basis of inhibitory control as a function of the Social context and Cue type

Post-hoc analyses revealed that during inhibitory control (Stop Correct > Stop Incorrect) CUD participants showed higher activity in the right OFC, left OFC, and right IFG for cocaine cues compared to neutral cues in the non-observer condition (all *Ps* < 0.01). Interestingly, they showed an opposite pattern in the observer condition (Fig. 4). Here, there was lower brain activity within these regions for cocaine cues compared to neutral ones (all *Ps* < 0.040). This shift in brain activity patterns in the observer condition was driven by both a lower response to cocaine cues in comparison to the non-observer condition (all *Ps* < 0.028) and a higher response to neutral cues (all *Ps* < 0.036). No other significant results emerged. Likewise, CUD participants exhibited lower right STN activity during inhibition when exposed to cocaine cues versus neutral cues in the observer condition (*p* = 0.012). This difference was primarily due to higher right STN activity in response to neutral stimuli when comparing the observer condition to the non-observer condition (*p* = 0.032) (Fig. 3). Moreover, in participants with CUD, the left MTG exhibited a different response pattern compared to the previously mentioned brain regions. Specifically, in the non-observer condition, CUD participants showed lower left MTG activity for cocaine cues compared to neutral cues (*p* < 0.001). Conversely, in the observer condition, there was higher left MTG activity for cocaine cues relative to neutral cues (*p* < 0.001). This change was attributed to both higher left MTG activity for cocaine cues when comparing observer to non-observer conditions (*p* < 0.001) and lower activity for neutral cues in the observer condition compared to the non-observer condition (*p* < 0.001). No other significant results emerged. Regarding the HC group, analysis revealed higher activity in the right OFC during inhibition in the presence of cocaine cues as opposed to neutral cues in the observer condition (*p* = 0.008), while no difference emerged between these cues in the non-observer condition (*p* = 0.459). Additionally, when comparing the observer condition to the non-observer condition, there was lower right OFC activity in the presence of neutral cues (*p* = 0.036). No difference between observer versus non-observer conditions was found for cocaine-cues for HC participants (*p* = 0.214). Also, no significant results for HC participants emerged for the left OFC (all *Ps* > 0.15). Furthermore, HC participants exhibited lower activity in the right IFG when exposed to cocaine cues versus neutral cues in the non-observer condition (*p* = 0.008). No other significant results emerged.

Exact parameter estimates, *t*- and *p*-values are provided in the Supplementary Materials (Tables S1–S5). Our findings suggest that CUD participants tend to exhibit distinct inhibitory control brain processing depending on the cue type. Notably, this effect appears to be significant and in the opposite direction when an observer is present. See Fig. 3 and Fig. 4 (and see Fig. S1 and Fig. S2 for neuroimaging results for each group, separately).

#### Differential effects of cue type and social context on inhibitory control: higher changes in CUD participants compared to HC

During inhibitory control (Stop Correct > Stop Incorrect), individuals with CUD showed significant higher brain activity in the right OFC and right IFG compared to HC in the presence of cocaine cues in the non-observer condition (all *Ps* < 0.05). Interestingly, no differences in the right IFG activity emerged anymore between the two groups for cocaine cues when being observed by another person (*p* = 0.093) and even an opposite pattern of activity emerged in the right OFC. Specifically, participants with CUD showed lower activity in this brain area for cocaine cues when being observed, compared to HC (*p* = 0.007). As regards neutral cues, CUD participants showed lower activity in the right and left OFC as well as in the right IFG compared to HC participants (all *Ps* < 0.046) in the non-observer condition. Interestingly, in the observer condition, no group differences emerged for neutral cues in the left OFC and right IFG (all *Ps* > 0.069), while the right OFC showed even an opposite pattern. In particular, CUD participants showed a higher right OFC activity for neutral cues compared to HC in this condition (*p* = 0.012). Lastly, CUD participants showed lower activity in the left MTG during inhibition in the presence of cocaine cues compared to HC when not observed (*p* = 0.015) while no group differences emerged in the observer condition (*p* = 0.117). As regards neutral cues, CUD participants showed higher activity in the left MTG compared to HC when not observed (*p* = 0.018) and lower activity compared to HC when being observed (*p* = 0.034). Exact parameter estimates, *t*- and *p-*values are provided in the Supplementary Materials (Tables S1–S5).

#### Craving for cocaine and SSRT are associated with STN and OFC activities during inhibitory control, respectively

Among participants with CUD, there was a significant positive correlation between craving for cocaine (CCQ scores) and activity in the right STN during cocaine-related inhibitory control in the non-observer condition (*r* = 0.69, *p* = 0.003) (Fig. 3C) but not in the observer one (*p* = 0.76). In addition, the STN activity during cocaine-related inhibitory control (coc vs neu) in the observer condition positively correlated with the quantity of cocaine consumed per instance (estimated in grams) (rho = 0.61; *p* = 0.009) in CUD participants. This correlation was not significant in the non-observer condition (*p* = 0.122). Moreover, in the non-observer condition, better inhibitory performance in CUD participants was significantly associated with higher activity in the left OFC (*r* = −0.49, *p* = 0.044) (Fig. S3) and marginally associated in the right OFC (*r* = −0.48, *p* = 0.053). These correlations were not significant when participants were being observed (*Ps* > *0.30)*. In addition, we found a significant positive correlation between the left MTG activity and dependence severity according to DSM-V criteria (rho = 0.56; *p* = 0.019) in the observer condition and a significant negative correlation between these two variables in the non-observer condition (rho = −0.54; *p* = 0.025) in CUD participants. No other significant correlations emerged (all *Ps* > 0.067). No significant correlations were found in the HC group. Lastly, linear regression analyses revealed that higher activity in the right OFC during cocaine-related inhibitory control (across observer/non-observer conditions) was positively associated with higher activity in the right STN for both the CUD (*β* = 0.488, *p* = 0.003) and HC groups (*β* = 0.487, *p* = 0.003).

## Discussion

By combining fMRI and a social version of the Stop-Signal Task, we investigated whether the control of inhibition in cocaine addiction can be affected by either cocaine-related cues and/or the social context and which are the neural correlates of this possible modulation.

Our study demonstrates that individuals with CUD exhibit a reduced ability to inhibit their responses in the SST, particularly when analyzing only trials associated with neutral cues to align with previous research that utilized traditional SST paradigms. This finding is in line with research showing impaired inhibitory control in the SST for individuals with SUD [71–73]. However, other studies found no significant differences in SST performance between SUD participants and controls [74–77], and one study even reported enhanced inhibitory control performance in CUD participants compared to control participants for neutral and food cues in a novel version of the SST [46]. Furthermore, a study on heroin use disorder found no differences in SSRT compared to healthy controls but revealed reduced target detection sensitivity (proportion of hits in go vs false alarms in stop trials) in heroin users, indicating diminished perceptual sensitivity during inhibitory control [45]. This variability of findings could be attributed to several factors, including differences in study design, sample size, stages of SUD, specific abused substance, comorbidities, and the specific measures of inhibitory control employed. Though behavioral findings are varied, evidence for altered inhibitory control in SUD mainly comes from alterations in underlying neural processing (e.g., Impaired Response Inhibition and Salience Attribution (iRISA) model) [44].

Importantly, the engagement in seeking and taking drugs also depends upon the relative strength of the motivation or craving to use the drug [57, 78], which can impact a person’s ability to control their impulses [42, 44]. To test the potential modulation of inhibitory control by drug cue salience, we included for the first-time cocaine-related cues (using images) in an SST.

Interestingly, cocaine-related cues helped individuals with CUD to exhibit an improved inhibitory control compared to trials with neutral cues, reaching the level of HC. In contrast, HC did not show any difference in performance between cocaine and neutral-related trials, indicating that cocaine cues only affect inhibitory control in individuals with CUD and not in HC (possibly because drug cues were not salient or relevant for the HC). It might be surprisingly that cocaine cues did not impair the stopping performance of CUD participants, but rather improved it. One possible explanation for this finding is that these cues elicited craving for cocaine, potentially increasing motivation, arousal, and attentional focus on these salient trials specifically for CUD participants. Indeed, our neuroimaging results show that the more CUD participants experienced craving for reward, as measured by the CCQ questionnaire, the greater the activity in their right STN during cocaine-related inhibitory control.

Previous studies have shown the crucial role of the STN in inhibitory control processes, as a key component of the basal ganglia-thalamocortical circuit involved in motor and cognitive control [29–31, 33]. Also, the STN encodes both drug and natural reward values [18, 39, 79, 80]. Thus, our findings suggest that the presence of drug cue stimuli during an inhibitory task such as the SST can activate the STN, leading to increased inhibitory control, since STN inhibition reduces it [32, 33, 78].

Importantly, the STN receives direct inputs from cortical regions such as the IFG and the orbitofrontal cortex OFC via the hyperdirect pathway. It has been shown that these cortico-STN hyperdirect pathways facilitate the suppression of the ongoing motor response [29, 34, 35]. Strikingly, our study revealed exactly the engagement of these brain regions during inhibitory control. In particular, CUD participants exhibited higher activity in these regions during inhibition in trials involving cocaine-cues compared to those with neutral cues. Conversely, HC participants showed heightened activity in these brain areas in trials involving neutral cues relative to cocaine cues. Our finding suggests that salient drug cues in addiction can modulate the activity of these frontal regions, which in turn may influence the STN, possibly via a network that regulates inhibitory control processes. This finding is further corroborated by the negative association that we found between the OFC activity during cocaine-related inhibitory control and CUD participants’ SSRT, showing that a higher activity in this brain area was associated with a better inhibition in trials with cocaine-cues. By highlighting the involvement of both OFC and STN in the regulation of the inhibition, our data confirm the network underscored in a review [33].

As regards the impact of the presence of an observer on inhibitory control, we found a modulation of the activity of the OFC, IFG, and STN in CUD participants under this condition. This change in brain activity patterns in the observer condition was driven by both a lower response to cocaine cues in comparison to the non-observer condition and a higher response to neutral cues. The lower activity for cocaine cues in presence of an observer could be interpreted as a competition for processing resources between the potential reward of the social interaction and the drug cue. One might have expected an additivity of rewarding situations such as cocaine cue and social presence, but the lack of additivity suggests an interaction between these processes within the STN. Notably, such competition seems to be cocaine dependent since neither the cues nor the social presence influenced the HC group, potentially because social presence does not compete with a pre-existing strong association with the cocaine cues (unlike CUD participants). It is important to acknowledge that this interpretation is primarily inspired by animal studies that have shown reduced cocaine intake in presence of a peer or playback of ultrasonic vocalizations and also that physical presence or ultrasonic vocalizations act as an alternative reward since they can be “self-administered” or induce conditioned place preference [11, 16, 17, 81]. This requires further investigation in humans, although the presence of a peer has been reported to reduce cocaine consumption [11]. Furthermore, it is worth noting that our study also revealed an interesting finding regarding the association between craving for cocaine, neural activity in the STN during cocaine-related inhibitory control, in line with the former findings showing that inhibition of the STN can reduce motivation for cocaine or escalation of cocaine intake in rats [18–20] and the relationship between OFC activity and stopping abilities in individuals with CUD. Importantly, these associations were significant only in the non-observer condition. This further suggests that the presence of an observer may dampen these associations, in line with the modulations observed after STN lesions in rats self-administering cocaine in presence of a peer [11]. Moreover, STN activity during cocaine-related inhibitory control correlates with drug intake in presence of the observer, further corroborating this possible interaction between cocaine cue and social context at the level of the STN. Showing that activity of STN is modulated by the presence of a peer confirm the critical role of STN in the reduction of cocaine intake observed when subjects (rats like human) are in presence of a peer [11].

Another neural signature of cocaine-related inhibitory control is the higher activity in the left MTG, when CUD participants were observed compared to when they were not. This difference was not found in HC participants. Also, when not under observation, individuals with CUD exhibited lower left MTG activity as compared to HC. However, when observed by another person, CUD participant showed higher left MTG activity compared to HC. The MTG is involved in various cognitive processes, including attention and perception, both often in relation to social cognitive processes [27, 82–84]. This heightened activity in the MTG could be a result of increased attention and vigilance triggered by the awareness of being observed. It may reflect anticipatory and evaluative processes where individuals focus on their actions and potential consequences in a social context. Interestingly, the left MTG activity during cocaine-related inhibitory control was associated with severity of dependence in CUD participants. This relationship changes depending on the presence or absence of an observer and suggests the potential role of the left MTG in mediating social-attentional processes during inhibitory control in cocaine addiction.

Also, it is important to note that our study used the experimenter wearing a labcoat as the observer. Thus, the observer was a non-familiar person to the participant. Future human studies should explore the use of familiar peers on control of inhibition in CUD, as well as peers who are either drug-naïve or drug users, as this could yield different outcomes. Indeed, in a previous study on rats and human cocaine users [11], the drug consumption was reduced depending on the type of the peer, with a strong effect when a peer was present, abstinent, or drug-taking as well, further diminished when the peer was non-familiar. Despite being confident on the significant results reported in the manuscript, in particular the three-way interaction, the limited sample size in the current study precludes any conclusion on the non- significant effects. Another limitation of our study is the absence of assessments for intelligence quotient (IQ) or other neuropsychological functions (e.g., executive functions like working memory), that may be mediated by the prefrontal cortex. Furthermore, because the SSST employed in this study only compares between a drug and neutral cue, it may not be possible to rule out potential arousal effects, which would be accomplished by using stimuli that are matched in arousal, like a nondrug reward (i.e., a food reward) [46, 57]. Future research on substance abuse might include stimulus ratings measuring valence and arousal in order to control for these possible confounding effects.

Given that social interactions are inherently reciprocal, and recent research suggests that brain and cognitive processes differ in interactive (second-person) versus observational (third-person) contexts [25, 85, 86], future research could use even more naturalistic situations to investigate the effect of the social context on inhibitory control.

To the best of our knowledge, this is the first study assessing the brain systems that regulate the complex interplay between drug cues, social factors, and inhibitory control in cocaine addiction in humans. Our findings can shed light on potential targets for intervention and suggest the importance of considering further the social context in addiction research and treatment. Given that there is still space for improvement in the management of cocaine-related disorders, these results may be crucial to developing harm reduction strategies for cocaine users.

## Supplementary information

Supplementary Materials

## Data Availability

The raw neuroimaging data cannot be openly shared due to local legal restrictions. Anonymized behavioral data will be available at https://osf.io/q359w/?view_only=76f811aa4434458f8415aa42bfbc4a6f.

## Code availability

All codes are available upon request.
