# Supplementary material for: Social context and drug cues modulate inhibitory control in cocaine addiction: involvement of the STN evidenced through functional MRI
Source: Mol Psychiatry. 2024 Jun 26;29(12):3742–51. doi: 10.1038/s41380-024-02637-y (PMC11609098; doi:10.1038/s41380-024-02637-y)
Supplement: Supplementary file 1 — Supplementary Materials [file 41380_2024_2637_MOESM1_ESM.doc]

**Supplementary Information**

**Social Context and Drug Cues Modulate Inhibitory Control in Cocaine Addiction: involvement of the STN evidenced through Functional MRI**

Damiano Terenzi^1*^, Nicolas Simon^1,2^, Michael Joe Munyua Gachomba^1^, Jeanne Laure de Peretti^1^, Bruno Nazarian^1^, Julien Sein^1^, Jean-Luc Anton^1^, Didier Grandjean^3^, Christelle Baunez^1*#^, and Thierry Chaminade^1#^

*** corresponding author**

**To whom correspondence may be addressed: * EMAIL: damiano.TERENZI@univ-amu.fr; christelle.baunez@cnrs.fr**

#These authors contributed equally

^1^ Institut de Neurosciences de la Timone, UMR 7289 CNRS & Aix-Marseille Université, Marseille, France

^2^ SESSTIM INSERM, IRD & Aix-Marseille Université, AP-HM, Marseille, France

^3^ Swiss Center for Affective Science and Department of psychology and educational sciences, University of Geneva, Switzerland

**Neuroimaging results: effects at the group level (individually for each group)**


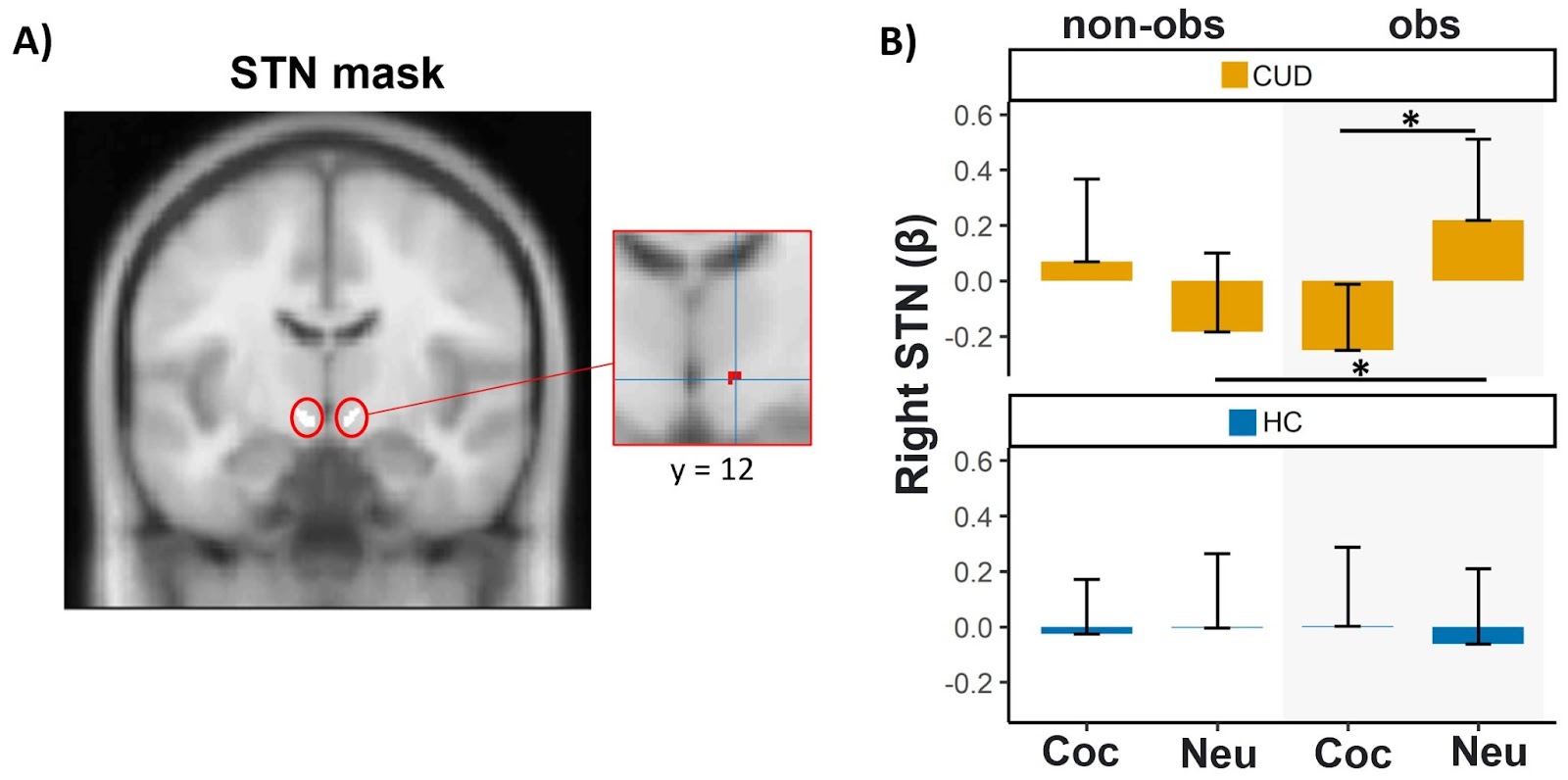


Figure S1. Brain activations related to inhibitory control for cocaine and neutral cues in presence or absence of an observer. Separate panels are presented for CUD (top) and HC (bottom) participants. A) The left image depicts the bilateral STN mask defined by [75]. The right image is a zoom-in inset showing significant Group X Social Context X Cue-type interaction [χ^2^ (1) = 4.82, p = 0.028] within the right STN mask for the contrast Stop Correct > Stop Incorrect. B) Bar graphs showing relative right STN activity to cocaine and neutral cues in CUD and HC participants when being observed compared to when they were not. Higher values indicate increased STN activity during inhibition. The error bars represent the standard error to mean SEM . *= p < 0.05.


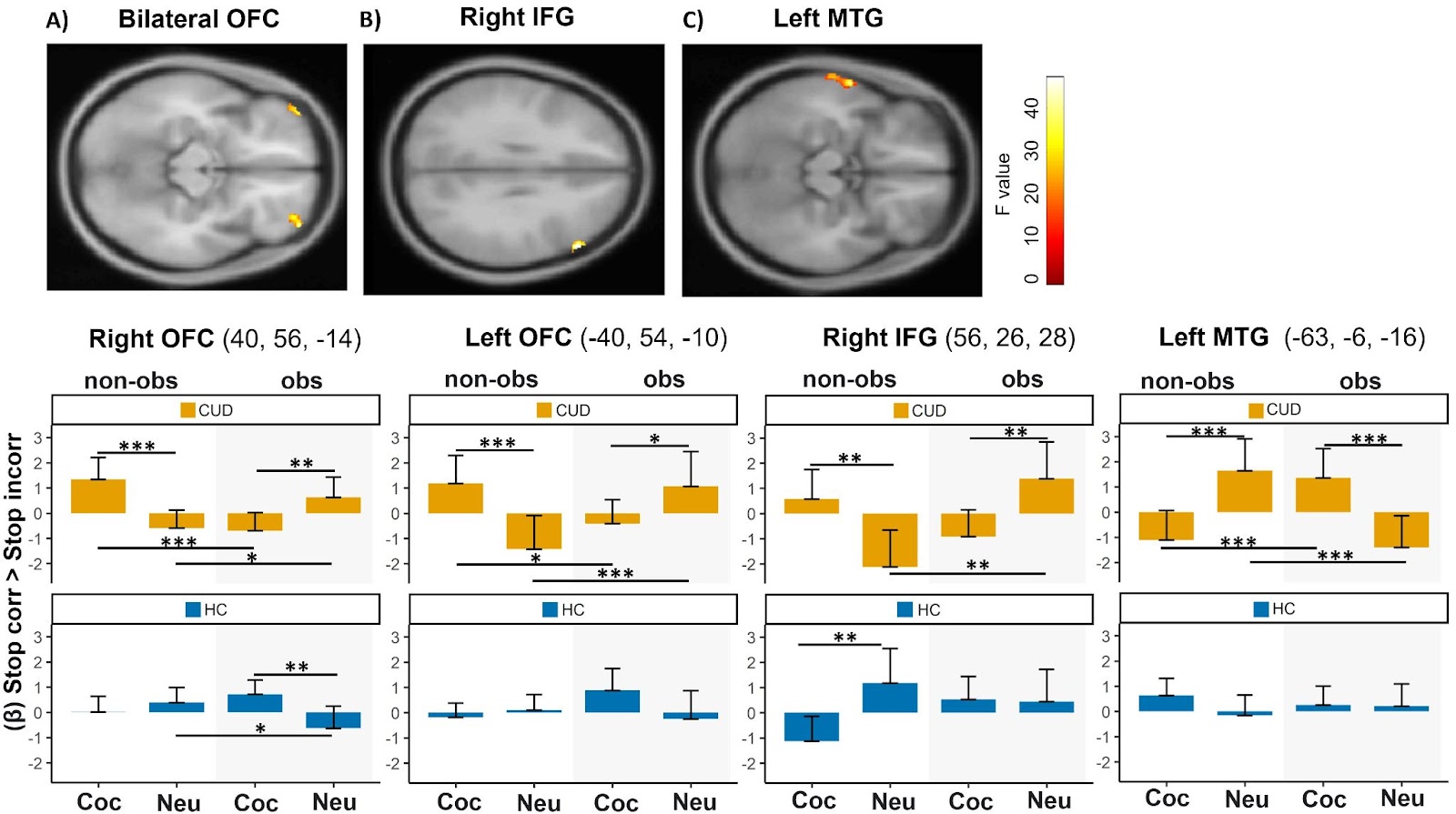


Figure S2. Top: Brain activations related to inhibitory control for cocaine (Coc) and neutral (Neu) cues in presence (obs) or not (non-obs) of an observer. Whole brain results showing significant Group X Social Context X Cue-type interactions in the left and right OFC (A), right IFG (B), and left MTG (C) (P_FWE_, < 0.05) for the contrast Stop Correct > Stop Incorrect. Bottom: The bar graphs represent mean signal intensity from these significant interaction clusters (Error bar SEM). Higher values indicate increased activity during inhibitory control. Separate panels are presented for CUD (top; orange bars) and HC (bottom; blue bars) participants *= p < 0.05; **= p < 0.01; ***p < 0.001.

**Social context manipulation instructions verbatim**

The instruction was partially scripted, but this was hidden from the participants by choosing a verbal presentation of the experimental paradigm by the experimenter acting as the observer (TC) dressed and hair combed as in the videos purposely presented live during the actual scanning. For all participants the experimental procedure was presented as a three step process:

First, we present the organization of a trial, with the cue apparition signaling the trial onset; after a random duration, an arrowhead (< or >) was superimposed indicating the right hand finger (index or major) that was to press the key as quickly as possible.

Second, we introduced the inhibitory signal, a sound indicating that the response should be inhibited, and explained that the duration between cue and arrowhead wasn't random, but actually adapted to make the task easier or harder in order for the participants to achieve correct responses in approximately half of the trials. This was important to avoid frustration from failure in the context of adaptive time-delay (ie, making explicit that it was part of the experimental procedures that mistakes would be made) as well as to ensure they would not adopt an alternative strategy of waiting for the sound signal, that would increase their success rate but hinder the inhibitory control under investigation.

Third, after making sure the last aspect was apprehended, we explained the control blocks in which the sound cue should be neglected altogether. The overall organization of the recording sessions (4 sessions, approximately 7 minutes per session, number of experimental and control blocks) was presented together as an active webcam filming the experimenter facing the control computer with the following sentence "Sometimes I (the experimenter) will check your performance in the task by monitoring your responses and you will see me on the webcam live flow inlaid on the top-left corner of the image you're seeing usually used to monitor patients". This presentation was purposely chosen to ensure participants believed that the live verification of their behavior was incidental and not an experimental procedure, to enforce the idea of a live (*versus* a scripted) setup.

**No significant associations between education level and behavioral and neuroimaging results**

We explored the potential association of education level with the behavior (SSRT) and neuroimaging results (the brain areas identified from the whole-brain fMRI results through a three-way interaction, as well as the STN region of interest results) for both the observer/non-observer conditions and for both groups. Spearman correlations revealed no significant associations for both CUD (all Ps > 0.169) and HC (all Ps > 0.068) participants.

**Tables S1-S5**

| **Comparison – right STN** | **Contrast** | **Estimate** | **t-ratio** | **p-value** |
| --- | --- | --- | --- | --- |
| HC, COC | non-obs vs. obs | -0.059 | -0.317 | 0.7513 |
| CUD, COC | non-obs vs. obs | 0.323 | 1.751 | 0.0813 |
| HC, NEU | non-obs vs. obs | 0.029 | 0.161 | 0.8725 |
| CUD, NEU | non-obs vs. obs | -0.398 | -2.161 | **0.0317** |
| HC vs. CUD, COC (non-obs) | HC vs. CUD | -0.112 | -0.592 | 0.5543 |
| HC vs. CUD, COC (obs) | HC vs. CUD | 0.270 | 1.421 | 0.1567 |
| HC vs. CUD, NEU (non-obs) | HC vs. CUD | 0.164 | 0.867 | 0.3871 |
| HC vs. CUD, NEU (obs) | HC vs. CUD | -0.264 | -1.393 | 0.1652 |
| HC, COC vs. NEU (non-obs) | COC vs. NEU | -0.022 | -0.123 | 0.9023 |
| HC, COC vs. NEU (obs) | COC vs. NEU | 0.066 | 0.359 | 0.7202 |
| CUD, COC vs. NEU (non-obs) | COC vs. NEU | 0.254 | 1.377 | 0.1697 |
| CUD, COC vs. NEU (obs) | COC vs. NEU | -0.467 | -2.535 | **0.0119** |

| **Comparison – right OFC** | **Contrast** | **Estimate** | **t-ratio** | **p-value** |
| --- | --- | --- | --- | --- |
| HC, COC | non-obs vs. obs | -0.638 | -1.247 | 0.2138 |
| CUD, COC | non-obs vs. obs | 2.030 | 3.996 | **0.0001** |
| HC, NEU | non-obs vs. obs | 1.082 | 2.114 | **0.0356** |
| CUD, NEU | non-obs vs. obs | -1.224 | -2.409 | **0.0168** |
| HC vs. CUD, COC (non-obs) | HC vs. CUD | -1.28 | -2.522 | **0.0123** |
| HC vs. CUD, COC (obs) | HC vs. CUD | 1.38 | 2.718 | **0.0071** |
| HC vs. CUD, NEU (non-obs) | HC vs. CUD | 1.02 | 2.006 | **0.0461** |
| HC vs. CUD, NEU (obs) | HC vs. CUD | -1.28 | -2.522 | **0.0124** |
| HC, COC vs. NEU (non-obs) | COC vs. NEU | -0.377 | -0.742 | 0.4587 |
| HC, COC vs. NEU (obs) | COC vs. NEU | 1.343 | 2.642 | **0.0088** |
| CUD, COC vs. NEU (non-obs) | COC vs. NEU | 1.929 | 3.796 | **0.0002** |
| CUD, COC vs. NEU (obs) | COC vs. NEU | -1.326 | -2.609 | **0.0097** |

| **Comparison – left OFC** | **Contrast** | **Estimate** | **t-ratio** | **p-value** |
| --- | --- | --- | --- | --- |
| HC, COC | non-obs vs. obs | -1.043 | -1.442 | 0.1506 |
| CUD, COC | non-obs vs. obs | 1.588 | 2.212 | **0.0280** |
| HC, NEU | non-obs vs. obs | 0.367 | 0.507 | 0.6124 |
| CUD, NEU | non-obs vs. obs | -2.493 | -3.472 | **0.0006** |
| HC vs. CUD, COC (non-obs) | HC vs. CUD | -1.35 | -1.875 | 0.0621 |
| HC vs. CUD, COC (obs) | HC vs. CUD | 1.28 | 1.775 | 0.0772 |
| HC vs. CUD, NEU (non-obs) | HC vs. CUD | 1.53 | 2.126 | **0.0346** |
| HC vs. CUD, NEU (obs) | HC vs. CUD | -1.33 | -1.842 | 0.0668 |
| HC, COC vs. NEU (non-obs) | COC vs. NEU | -0.285 | -0.397 | 0.6919 |
| HC, COC vs. NEU (obs) | COC vs. NEU | 1.125 | 1.567 | 0.1185 |
| CUD, COC vs. NEU (non-obs) | COC vs. NEU | 2.600 | 3.620 | **0.0004** |
| CUD, COC vs. NEU (obs) | COC vs. NEU | -1.482 | -2.064 | **0.0401** |

| **Comparison – right IFG** | **Contrast** | **Estimate** | **t-ratio** | **p-value** |
| --- | --- | --- | --- | --- |
| HC, COC | non-obs vs. obs | -1.657 | -1.918 | 0.0563 |
| CUD, COC | non-obs vs. obs | 1.486 | 1.733 | 0.0845 |
| HC, NEU | non-obs vs. obs | 0.743 | 0.860 | 0.3904 |
| CUD, NEU | non-obs vs. obs | -3.505 | -4.086 | **0.0001** |
| HC vs. CUD, COC (non-obs) | HC vs. CUD | -1.694 | -1.971 | **0.0499** |
| HC vs. CUD, COC (obs) | HC vs. CUD | 1.449 | 1.686 | 0.0932 |
| HC vs. CUD, NEU (non-obs) | HC vs. CUD | 3.306 | 3.846 | **0.0002** |
| HC vs. CUD, NEU (obs) | HC vs. CUD | -0.942 | -1.096 | 0.2743 |
| HC, COC vs. NEU (non-obs) | COC vs. NEU | -2.3081 | -2.691 | **0.0076** |
| HC, COC vs. NEU (obs) | COC vs. NEU | 0.0924 | 0.108 | 0.9143 |
| CUD, COC vs. NEU (non-obs) | COC vs. NEU | 2.6924 | 3.139 | **0.0019** |
| CUD, COC vs. NEU (obs) | COC vs. NEU | -2.2986 | -2.680 | **0.0079** |

| **Comparison – left MTG** | **Contrast** | **Estimate** | **t-ratio** | **p-value** |
| --- | --- | --- | --- | --- |
| HC, COC | non-obs vs. obs | 0.471 | -0.678 | 0.4987 |
| CUD, COC | non-obs vs. obs | -2.477 | -3.590 | **0.0004** |
| HC, NEU | non-obs vs. obs | -0.271 | -0.391 | 0.6965 |
| CUD, NEU | non-obs vs. obs | 3.034 | 4.397 | **<.0001** |
| HC vs. CUD, COC (non-obs) | HC vs. CUD | 1.79 | 2.451 | **0.0152** |
| HC vs. CUD, COC (obs) | HC vs. CUD | -1.15 | -1.577 | 0.1166 |
| HC vs. CUD, NEU (non-obs) | HC vs. CUD | -1.74 | -2.383 | **0.0182** |
| HC vs. CUD, NEU (obs) | HC vs. CUD | 1.56 | 2.133 | **0.0342** |
| HC, COC vs. NEU (non-obs) | COC vs. NEU | 0.788 | 1.143 | 0.2542 |
| HC, COC vs. NEU (obs) | COC vs. NEU | 0.046 | 0.067 | 0.9463 |
| CUD, COC vs. NEU (non-obs) | COC vs. NEU | -2.749 | -3.985 | **0.0001** |
| CUD, COC vs. NEU (obs) | COC vs. NEU | 2.761 | 4.003 | **0.0001** |

**SSRT results including all participants**

We performed a LMM investigating SSRT in correctly inhibited Stop trials. We limited our initial analyses to trials with neutral cues to maintain consistency with prior stop-signal task studies not using cocaine cues. This analysis showed a main effect of Group [χ^2^ (1) = 86.38, p = 0.028]. Post-hoc analysis showed slower SSRT (reduced inhibition) for users compared to controls (β = -37.26, t = -2.05, *p* = 0.048). No other significant effects of factors emerged (all *Ps* > 0.439). When performing a second LMM including all trial types (both neutral and cocaine cues), this model led to a significant two-way interaction Group x Cue type [χ^2^ (1) = 6.38, *p*= 0.011]. Post-hoc analysis showed that cocaine users had faster SSRT for cocaine-related trials compared to neutral ones (β = -22.24, t = -2.71, *p* = 0.035) (Figure 2). This result persisted even when controlling for participants’ educational level (β  = -22.23, t = -2.71, *p* = 0.035). No other significant results emerged.


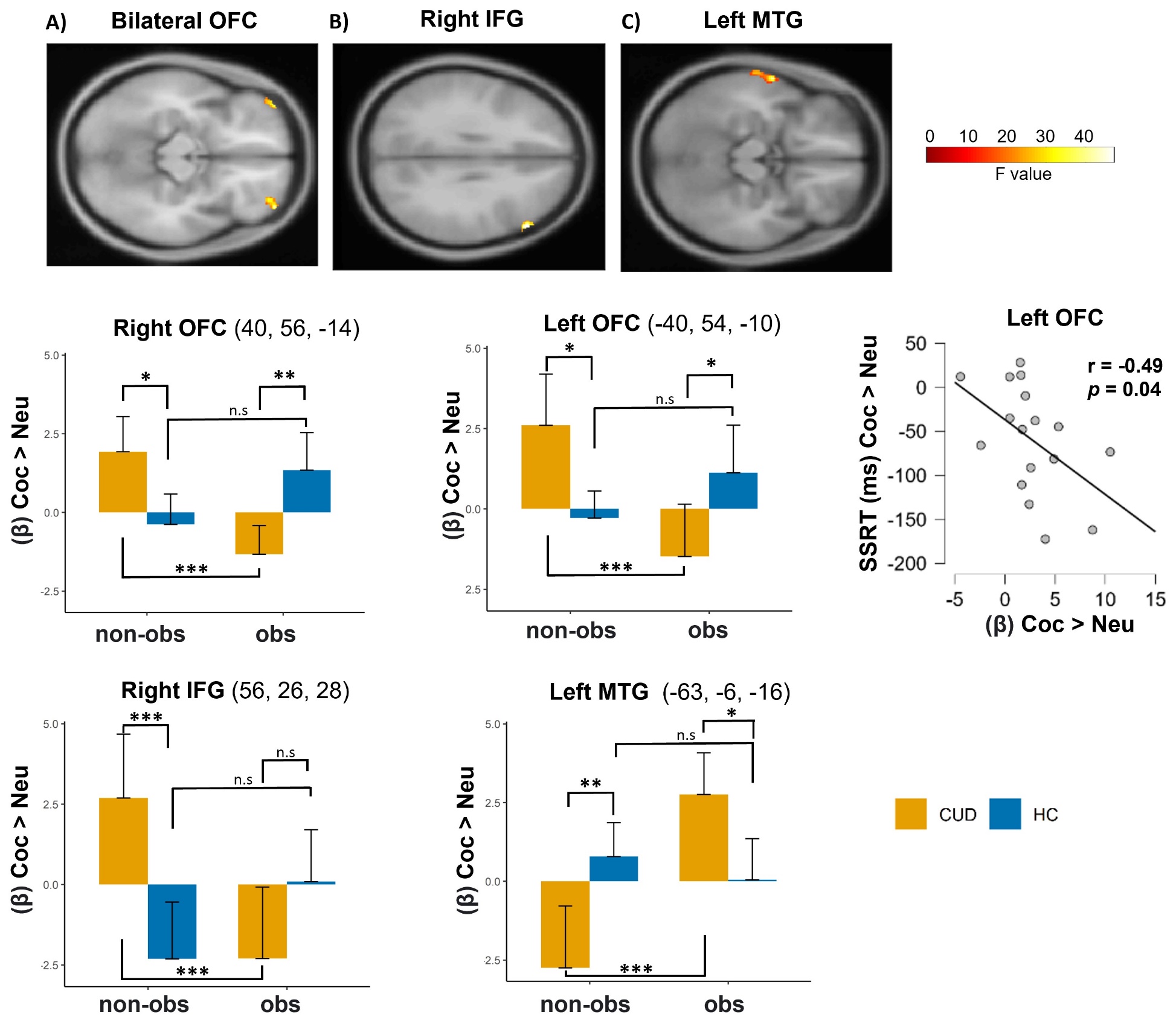


**Figure S3**. The scatterplot represents the significant negative correlation between CUD participants’ SSRT (cocaine > neutral) and left OFC activity for the same contrast (r = -0.49, p = 0.044). Lower values mean faster SSRT for cocaine compared to neutral-related cues.
